# Transcriptional activation of the Axl and PDGFR-α by c-Met through a ras- and Src-independent mechanism in human bladder cancer

**DOI:** 10.1186/1471-2407-11-139

**Published:** 2011-04-16

**Authors:** Chen-Yun Yeh, Shin-Mei Shin, Hsuan-Heng Yeh, Tsung-Jung Wu, Jyh-Wei Shin, Tsuey-Yu Chang, Giri Raghavaraju, Chung-Ta Lee, Jung-Hsien Chiang, Vincent S Tseng, Yuan-Chii G Lee, Cheng-Huang Shen, Nan-Haw Chow, Hsiao-Sheng Liu

**Affiliations:** 1 Department of microbiology and immunology, College of medicine, National Cheng Kung University, Tainan, Taiwan; 2 Department of pathology, College of medicine, National Cheng Kung University, Tainan, Taiwan; 3 Institute of Basic Medical Sciences, National Cheng Kung University, Tainan, Taiwan; 4 Department of parasitology, College of medicine, National Cheng Kung University, Tainan, Taiwan; 5 Department of computer science and information engineering, National Cheng Kung University, Tainan, Taiwan; 6 Center for gene regulation and signal transduction research, National Cheng Kung University, Tainan, Taiwan; 7 Graduate Institute of Medical Informatics, Taipei Medical University, Taipei, Taiwan; 8 Department of Urology, Chia-Yi Christian Hospital, Chia-Yi, Taiwan

**Keywords:** Axl, PDGFR-α, c-Met, bladder cancer

## Abstract

**Background:**

A cross-talk between different receptor tyrosine kinases (RTKs) plays an important role in the pathogenesis of human cancers.

**Methods:**

Both NIH-Met5 and T24-Met3 cell lines harboring an inducible human c-Met gene were established. C-Met-related RTKs were screened by RTK microarray analysis. The cross-talk of RTKs was demonstrated by Western blotting and confirmed by small interfering RNA (siRNA) silencing, followed by elucidation of the underlying mechanism. The impact of this cross-talk on biological function was demonstrated by Trans-well migration assay. Finally, the potential clinical importance was examined in a cohort of 65 cases of locally advanced and metastatic bladder cancer patients.

**Results:**

A positive association of Axl or platelet-derived growth factor receptor-alpha (PDGFR-α) with c-Met expression was demonstrated at translational level, and confirmed by specific siRNA knock-down. The transactivation of c-Met on Axl or PDGFR-α *in vitro *was through a *ras*- and Src-independent activation of mitogen-activated protein kinase/extracellular signal-regulated kinase (MEK/ERK) pathway. In human bladder cancer, co-expression of these RTKs was associated with poor patient survival (*p *< 0.05), and overexpression of c-Met/Axl/PDGFR-α or c-Met alone showed the most significant correlation with poor survival (*p *< 0.01).

**Conclusions:**

In addition to c-Met, the cross-talk with Axl and/or PDGFR-α also contributes to the progression of human bladder cancer. Evaluation of Axl and PDGFR-α expression status may identify a subset of c-Met-positive bladder cancer patients who may require co-targeting therapy.

## Background

The RTK c-Met is expressed during normal development and plays a crucial role in many cell regulatory processes [1]. After binding to its cognate ligand-hepatocyte growth factor (HGF), activated c-Met transmits signals implicated in the cell proliferation, motility, survival, and morphogenesis [2-4]. C-Met is over-expressed and usually associated with metastatic progression of a variety of human malignant tumors, including bladder cancer [1,5]. We have reported that c-Met is over-expressed in 32.3%, 63.2%, and 65.2% of superficial, locally advanced and metastatic bladder cancer, respectively [6]. Over-expression of c-Met is positively associated with muscle invasion and poor long term survival (p < 0.001), while it is not related to patient outcome in the subset of superficial bladder cancer. Miyata *et al. *also reported the significance of c-Met in bladder cancer development and as an important predictor of metastasis and patient survival [7]. Therefore, c-Met is emerging as a novel therapeutic target in many solid tumors [8-10].

Dimerization is generally required for activating RTKs [11]. In addition to heterodimeric complex formation of the same subfamily [6,11-14], heterologous RTK interaction is also involved in the pathogenesis of human cancers, e.g. between EGFR and RON (a member of the c-Met family) [15,16]. The biological significance of inhibition of both RTK signaling pathways of cancer cells was demonstrated in the context of cell proliferation, migration, anti-apoptosis and transformation *in vitro*. [15]. Therefore, identification of cross-talk partners of c-Met involved in the tumorigenesis may provide important biomarkers for co-targeting therapy. In our prior RTK profiling experiment, c-Met was frequently co-expressed with Axl, platelet-derived growth factor receptor α (PDGFR-α), DDR2 and/or IGF1R in the same uroepithelial cells [17], suggesting the existence of yet unspecified cross-talk partners of c-Met.

Axl overexpression is detected in various human cancers, and is associated with invasiveness and/or metastasis of carcinoma of the breast [18], stomach [19], kidney [20], lung [21], and prostate [22]. High expression of PDGFR-α is also detected in a variety of tumors, such as prostatic intraepithelial neoplasia, and carcinoma of the ovary, kidney, breast and liver [23-26]. Furthermore, PDGFR-α expression provides additional predictive value related to breast cancer progression [23], and patient's survival in the kidney cancer [27] or lung cancer [28]. The implications of these two receptor-related signaling events in the bladder carcinogenesis, however, remain unclear. This study was aimed to identify the novel interaction partners of c-Met, investigate their regulation, effect on biological activity, and the potential significance in association with patient outcome.

## Methods

### Cell Lines, transfection, and stable cell line establishment

NIH/3T3 mouse fibroblast cell line and bladder cancer cell line T24 were obtained commercially. The four bladder cancer cell lines UB09: stage B2; UB40: stage A, papillary; UB47: stage B1; TSGH8301: stage A were established from patients of transitional cell carcinoma of the urinary tract [6,29]. UB47 was cultured in RPMI medium 1640 supplemented with 15% fetal bovine serum (FBS). Other cell lines were cultured in Dulbecco's modified Eagle's medium (DMEM) supplemented with 10% FBS.

The plasmids pTRE-Met and pTet-Lac-Hyg were transfected into NIH/3T3 and T24 cells by Lipofectamine™ 2000 reagent according to manufacturer's protocol (Invitrogen, Carlsbad, CA, USA) [30]. Two stable cell lines: NIH-Met5 and T24-Met3 were established.

### Microarray array

RNA was isolated using TRIzol reagent (GIBCO BRL, Gaithersburg, MD, USA), followed by mRNA purification using Oligotex™ mRNA kit (Qiagen, Valencia, CA, USA). RNA samples were reverse transcribed into cDNA fluorescently labeled either with Cy3 or with Cy5. The labeled cDNA was hybridized with a microarray cDNA chip containing 192 RTK genes [31]. Data were imported and normalized using MeV: MultiExperiment Viewer (Dana-Farber Cancer Institute, http://www.tm4.org/mev.html) [32]. Clustering affinity search technique (CAST) was used for gene expression cluster analysis. There are 23 clusters after CAST analysis [33], the gene expression profiles of 8 genes showing the best correlation with c-Met gene were clustered as one group (table 1).

**Table 1:** The mRNA expression levels of eight RTK genes clustered together with c-Met in RTK c-DNA microarray analysis

RTK name	NIH/3T3	NIH-Met5 day 1	NIH-Met5 day 4	NIH-Met5 day 7
**Axl**	-0.43	1.48	-0.71	-0.34
ERBB2	-0.24	1.34	-1.07	-0.02
ERBB3	-0.34	1.47	-0.79	-0.35
**Met**	0.02	1.18	-1.26	0.06
MST1R	-0.35	1.45	-0.84	-0.26
**PDGFRα**	-0.40	1.48	-0.70	-0.38
PDGFRβ	-0.20	1.13	-1.25	0.32
TIE1	-0.24	1.47	-0.76	-0.47
TIE2	-0.01	1.38	-0.97	-0.40

### Antibodies

Anti-phospho-tyrosine antibody was purchased from BD Transduction Laboratories (Lexington, KY, USA), and antibodies to Axl, c-Met, p-Met (phosphorylation of c-Met;Tyr 1234), PDGFR-α, and p-PDGFR-α (phosphorylation of PDGFR-α;Tyr 754) were purchased from Santa Cruz Biotechnology (Santa Cruz, CA, USA). The Ras antibody was obtained from Calbiochem (Merck, Darmstadt, Germany), Sp1 from Upstate Biotechnology Inc. (Golden, CO, USA), p-Axl (phosphorylation of Axl;Tyr 702) from Cell Signaling Technology Inc. (Beverly, MA, USA) and β-actin was purchased from Sigma-Aldrich (St. Louis, MO, USA). The Src antibody was obtained from Millipore (Billerica, MA, USA) and p-Src (phosphorylation of Src;Tyr 418**) **purchased from Invitrogen (Carlsbad, CA, USA).

### Western blot analysis

The western blot analysis was performed as previously described [6]. Briefly, the total lysates were prepared using RIPA solution. Total protein (50 μg) was analyzed by polyacrylamide gel electrophoresis and transferred to the PVDF membrane. The membrane was probed with targeted protein antibodies and the immune complex was detected with an enhanced chemiluminescence (ECL) detection system (Perkin Elmer Life Sciences, USA).

### siRNA transfection

Specific siRNA sense sequences were as follows: c-Met siRNA: 5`-**AAGTGCAGTATCCTCTGACAG**-3`, Axl siRNA: 5`-**CGTGGAGAACAGCGAGATTTA**-3`, and PDGFRα siRNA: 5`-**CGAGACGATTGATGCAGGATA**-3`. The cells (5 × 10^5^) were seeded into a 6-cm cell culture dish and incubated in DMEM medium without antibiotics. Lipofectamine 2000 reagent (10 μl) was diluted in 500 μl of DMEM serum-free media and incubated for 5 min at RT. The siRNA was diluted in 500 μl of DMEM serum-free medium to the assigned concentrations. Mock transfection was conducted in parallel using distilled water as the negative control. Then cells were incubated at 37°C in the 5% CO_2 _incubator for 4 h. The media were replaced with normal media and cells were incubated for additional 48 h before protein extraction.

### Trans-well migration assay

The effect of RTK cross-talk on cell migration was analyzed in TSGH8301 bladder cancer cells using a 24-well Transwell™ system (Corning inc., Lowell, MA). Briefly, cells were cultured in a 6-cm plate and transfected with c-Met, Axl, or PDGFR-α siRNA for 24 h, respectively. Then, cells were resuspended with serum-free medium and added into the upper chamber of the trans-well insert (2 × 10^5 ^cells/well). The 10% FBS-containing DMEM was added in the lower chamber. Cells were incubated h at 37°C for 36 h. Migrated cells were fixed with 4% formaldehyde in PBS and stained with 2% crystal violet in 2% ethanol. The non-migrated cells in the upper chamber were removed by wiping with a cotton swab. The cells on the lower surface of the filter, representing migration of the cells through the membrane, were counted under a light microscope.

### Clinicopathological characteristics of study cases

Since c-Met is important in the progression of bladder cancer, both locally advanced and metastatic bladder tumors were recruited to evaluate the significance of co-expression patterns of c-Met and other RTKs. Archival material of 65 patients (44 men and 21 women; age range, 40 to 84 yr old; mean ± SD, 61.5 ± 9.4 yrs) with locally advanced or metastatic urothelial bladder cancer (21pT2, 27pT3, 17pT4) was analyzed for RTK expression. These patients were diagnosed and treated in the National Cheng Kung University Hospital, Tainan, Taiwan, between 1990 and 1999 years. The numbers with low or high grade urothelial carcinoma were 20 and 45, respectively, according to definitions described previously [34]. Seven patients received partial cystectomy and remaining fifty-eight patients received radical cystectomy and bilateral pelvic lymph node dissection. Among them, 23 (35.4%) patients had pelvic lymph node involvement. An adjuvant systemic chemotherapy, including methotrexate, vinblastine, epirubicin, and cisplatin (M-VEC regimen), was given to 20 patients (30.8%) after radical cystectomy. The survival status was determined by outpatient clinic records and/or confirmed by interview with patients' families. Clinical follow-up ranged from 26 to 140 months (mean ± SD: 50.02 ± 6.46 months). The time of the first tumor recurrence and for disease specific survivals were counted. The time is calculated until the death of the patient due to bladder cancer. Patients who died of other causes or were still alive at the last follow-up were censored.

### Immunohistochemical staining

Immunostaining procedures were described in detail previously [6]. Briefly, tissue sections were incubated at RT for 2 h with monoclonal anti-cMet (1:100 dilution; Santa Cruz), anti-AXL (1:10 dilution; Santa Cruz) and anti-PDGFR-α (1:200 dilution; Santa Cruz) antibodies raised against the membrane protein. The optimal dilution was determined by using human kidney as a positive control [5]. Then StrAviGen Super Sensitive MultiLink kit (BioGenex Laboratories, Inc., San Ramon, California) was used to detect the resulting immune complex. Peroxidase activity was visualized using an aminoethyl carbazole substrate kit (Zymed Laboratories, Inc., San Francisco, Califonia).

Because no apparent difference of staining intensity was detected, only the proportion of tumor cells stained for c-Met, Axl or PDGFR-α was considered in classification [6]. "High level of expression" indicates > 50% of the tumor cells were immunostained, "low level of expression" indicates 10%-50% reactivity; and "negative" indicates < 10% staining for RTK protein.

### Statistical analysis

The association between tumor staging or gross characteristics with expression status of c-Met, Axl, and PDGFR-α was analyzed by Chi-square test as appropriate. The correlation between co-expression patterns of RTKs and disease-specific survival of cancer patients was constructed according to Kaplan-Meier method by Log rank test.

## Results

### Establishment of stable cell lines harboring inducible c-Met gene

**Figure 1:**
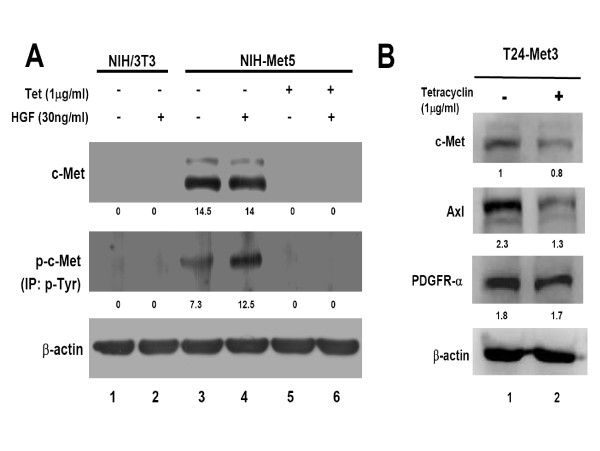
**c-Met expression in two stable cell lines in the presence or absence of Tet and/or HGF**. The total cell lysate (100 μg) from the treated cell lines was initially precipitated by the phosphorylated tyrosine antibody (p-Tyr), and then blotted with c-Met antibody to evaluate the phosphorylation of c-Met. The expression level of c-Met protein was measured by Western blotting. β-actin was used as an internal control. The intensity of each band in the gel was quantified by densitometry analysis using VisionWorks™ LS image acquisition and analysis software (UVP, USA) and labeled under each band. A: Both NIH/3T3 and NIH-Met5 cells were treated with or without Tet for 24 h, and then replaced with HGF (30 ng/ml) for 10 min. B: The stable human bladder cancer cell line T24-Met3 was maintained in the medium in the presence or absence of Tet for 24 h.

Two stable cell lines, designated as NIH-Met5 (mouse fibroblast) and T24-Met3 (human bladder cancer cell), were established to harbor the inducible c-Met gene, which was expressed only in the absence of tetracycline (Tet) (Figure 1A, lane 3 and 4; Figure 1B, lane 1). When c-Met was over-expressed, the increase of its phosphorylated form (p-Met) indicates an auto-phosphorylation (Figure 1A, lane 3; Additional file 1, lane 6). Expression of p-Met was further enhanced 10 min after treatment with hepatocyte growth factor (HGF; Sigma, St. Louis, MO, USA) (Figure 1A, lane 4; Additional file 1, lane 7). In contrast, parental NIH/3T3 cells did not express c-Met and p-Met (Figure 1A, lanes 1 and 2; Additional file 1, lane4 1-4). It is interesting to note that c-Met or p-Met was not expressed in NIH-Met5 cells when treated with Tet alone or combined with HGF treatment (Figure 1A, lanes 5 and 6; Additional file 1, lanes 8-9). Concerning T24-Met3 cells, expression of c-Met was suppressed 24 h after treatment with Tet (Figure 1B, lane 2). Together, auto-phosphorylation occurred when c-Met was over-expressed, and HGF treatment further enhanced the phosphorylation of c-Met. The results demonstrate a successful *in vitro *model in modulating the expression of c-Met using Tet-off system.

### Expression and functional association of c-Met with Axl and PDGFR-α in vitro

**Figure 2:**
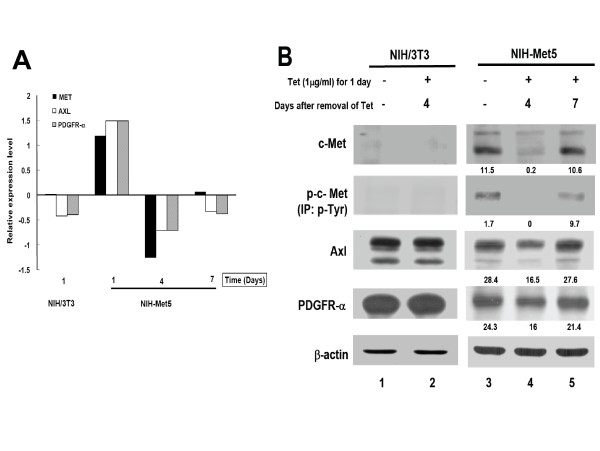
**Expression of c-Met, phosphorylated c-Met, Axl and PDGFR-α in NIH/3T3 and NIH-Met5 cells in the presence or absence of tetracycline**. A: A 192 RTK gene cDNA chip was used to screen the gene expression profiles which are positively correlated with c-Met in NIH/3T3 and inducible NIH-Met5 cell lines. A total of 8 RTK genes were shown to positively correlate with c-Met over-expression by CAST analysis (Methods). Only the gene expression profiles of c-Met, Axl and PDGFR-α are shown. NIH/3T3 (1D): the parental NIH/3T3 cell without Tet for 24 h; NIH-Met5 (1D): NIH-Met5 cell without Tet for 24 h; 4D: NIH-Met5 cell with Tet for 24 h followed by Tet-free treatment for another 4 days; 7D: NIH-Met5 cell with Tet for 24 h followed by Tet-free treatment for another 7 days. Relative expression level of each gene is obtained as compared with the common reference RNA [47] B: The expression levels of c-Met, p-Met, Axl and PDGFR-α were evaluated in NIH/3T3 and NIH-Met5 cells without Tet treatment (lanes 1 and 3), or after treatment for 24 h first and then replaced with Tet-free medium for additional 4 and 7 days, (lanes 4 and 5) by Western blotting. β-actin was used as an internal control. The numbers under each band represent the relative intensity.

To identify the novel interaction partners of c-Met, NIH-Met5 cells were first treated with Tet for 24 h, and then cultured in the absence of Tet for an additional 4 and 7 days (Figure 2A), respectively. Total RNA was extracted and subjected to screening using a cDNA microarray as previously described [35]. Among 192 RTKs, a total of 8 genes were positively correlated with c-Met over-expression, including Axl, PDGFR-α, PDGFR-β, ERBB2, ERBB3, MST1R, TIE1 and TIE2 (table 1). One of these candidate genes-MST1R was recently reported in our laboratory [6]. In addition, co-expression of c-Met with Axl and/or PDGFR-α was also detected in our pilot molecular profiling of RTKs in human bladder cancer cells *in vitro *[16]. As a result, both Axl and PDGFR-α were chosen for subsequent analysis. The comparable expression patterns of c-Met, Axl and PDGFR-α at RNA level were shown in Figure 2A.

The regulation was then examined at protein level in NIH-Met5 cells. As shown in figure 2B (lane 3), c-Met was overexpressed in the absence of Tet, while suppressed c-Met expression was demonstrated after treatment of Tet for 24 h, as with that of figure 1. A reversion of c-Met expression gradually appeared after removal of Tet for 4 and 7 days. Expression of c-Met became visible by day 4 and almost completely reversed by day 7 after removal of Tet (Figure 2B, lanes 4 and 5). The parental NIH/3T3 cells were used as a control (Figure 2B, lanes 1 and 2).

Using total c-Met and p-Met as the reference, expression of Axl and PDGFR-α showed a comparable trend to that of c-Met at day 4 and day 7 (Figure 2B, lanes 4 - 5), respectively. This positive association of Axl or PDGFR-α with c-Met expression was also demonstrated in T24-Met3 human bladder cancer cell line (Figure 1B). However, no difference of Axl and PDGFR-α expression was detected in NIH3T3 cells (Figure 2B, lanes 1 and 2). Taken together, expression patterns of total c-Met and p-Met were positively correlated with Axl and PDGFR-α expression, suggesting a functional relationship between Axl/PDGFR-α and c-Met.

### Correlation of c-Met expression with Axl and PDGFR-α status in human bladder cancer cells

**Figure 3:**
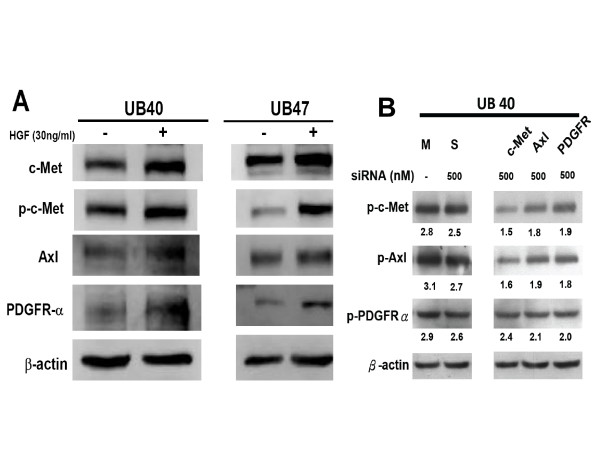
**Validation of the transactivation of c-Met, Axl and PDGFR-α in human bladder cancer cells**. A: Levels of c-Met, p-c-Met, Axl and PDGFR-α were measured in bladder cancer cell lines UB40 and UB47 by Western blotting after serum starvation for 24 h followed by HGF treatment for 10 min. B. c-Met, Axl and PDGFR-α protein expression was measured in UB40 cells after transfection with RTK-specific siRNAs (500 nM) by Western blotting. β-actin is a loading control. M: Mock transfection control. S: scramble siRNA (500 nM) is a negative control. The numbers under each band represent the relative intensity.

Both UB40 and UB47 are two bladder cancer cell lines established locally from primary bladder cancer of superficial and muscle-invasive type, respectively [6]. Apparent expression of c-Met and p-Met protein was detected in these two cell lines, and both Axl and PDGFR-α also showed a comparable expression pattern (Figure 3A). To confirm their functional interaction, these cell lines were maintained under serum starvation for 12 h, and then treated with HGF (30 ng/ml) for 10 min (Figure 3A). Up-regulation of Axl and PDGFR-α was demonstrated in UB40 and UB47 cells after HGF stimulation with a corresponding increase of p-Met (Figure 3A). Level of p-Met positively correlated with the expression of Axl and PDGFR-α, suggesting a relationship among c-Met, Axl and PDGFR-α.

To clarify the interaction among c-Met, Axl and PDGFR-α, UB40 cancer cells were transfected with c-Met, Axl and PDGFR-α specific siRNAs at the optimal concentrations for 48 h. When expression of each receptor protein was suppressed by their specific siRNA, expression levels of the other two proteins showed a trend of down-regulation, with a higher correlation between c-Met and Axl (Figure 3B). However, co-immunoprecipitation assay did not reveal evidence of direct interaction among these three RTK proteins at cell membrane level (data not shown). Taken together, the above data demonstrate a cross-talk among c-Met, Axl and PDGFR-α in a protein-protein interaction independent manner in human bladder cancer cells.

### The involvement of MEK/ERK signaling pathway in the transactivation of Axl and PDGFR-α by c-Met

**Figure 4:**
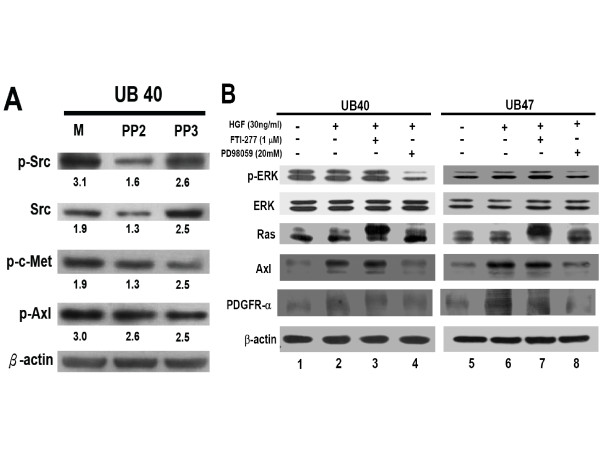
**The involvement of signaling regulation in the cross-talk of RTKs in human bladder cancer cells**. A: The UB40 cells were first pre-incubated with Src inhibitor (PP2) overnight. Then, phosphorylation status of Src, c-Met and Axl was evaluated after HGF stimulation for 10min. The PP3 (Calbiochem, Merck, Darmstadt, Germany) was used as a negative control for Src inhibition. The numbers under each band represent the relative intensity. B: After serum-starvation, UB40 and UB47 cells were treated with FTI-277 or PD98059 for 1 h, and then with HGF for 24 h. The p-ERK and levels of Ras, ERK, Axl and PDGFR-α expression were measured using specific antibodies by Western blotting. β-actin was used as an internal control. M: Mock transfection control.

There are several reports of signaling regulation about RTK transactivation. For example, a HGF-independent activation of c-Met by fibronectin was reported to promote the tumor invasion/metastasis [36]. Through binding to α_5_β_1_-integrin, fibronectin directly associates with c-Met and activates both Src and focal adhesion kinase activity. To clarify the potential involvement of this c-Met/Src-related signaling event, the Src inhibitor PP2 (Calbiochem, Merck, Darmstadt, Germany) was used to treat serum starved UB40 cells for 24 h. As shown in Figure 4A, suppression of Src phosphorylation did not alter the levels of c-Met and Axl, indicating that Src is not involved in the cross-talk of the three RTKs.

It is well known that MEK/ERK 1/2 is one of the most important transducer proteins when HGF binds to c-Met [1]. Both FTI-277 (a Ras farnesylation inhibitor; Calbiochem, San Diego, CA, USA) and PD98059 (a MEK 1 inhibitor; Cashmere Biotech Co., Taipei, Taiwan) were used to verify the involvement of MEK/ERK 1/2 signaling in c-Met-mediated activation of Axl and PDGFR-α. We showed that ERK phosphorylation was abrogated by PD98059 after HGF treatment for 24 h (Figure 4B, lanes 4 and 8) compared to FTI-277 (Figure 4B, lanes 3 and 7), suggesting the existence of a *ras*-independent phosphorylation of ERK mediated by HGF. The HGF-up-regulated Axl and PDGFR-α could be inhibited by PD98059 (Figure 4B, lanes 4 and 8), supporting the involvement of MEK/ERK 1/2 signaling in this transactivation event. In summary, MEK/ERK 1/2 signaling is involved in the transactivation of Axl and PDGFR-α by HGF/c-Met pathway in human bladder cancer cell lines, but is independent of *ras *or Src activity.

### The effect of cross-talk of c-Met, Axl, and PDGFR-α on cell migration

**Figure 5:**
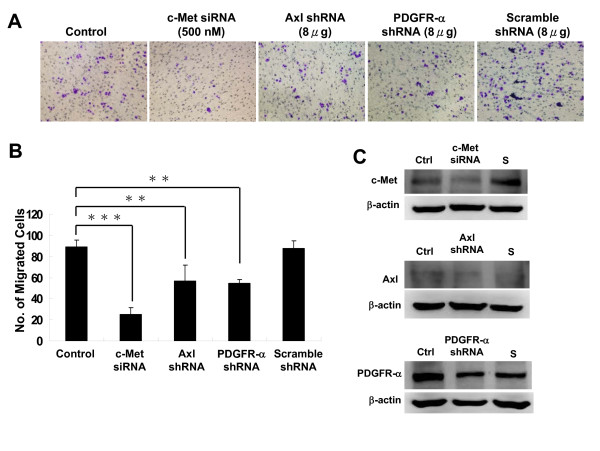
**The effect of c-Met, Axl and PDGFR-α expression on the migration of bladder cancer cells**. (A) TSGH8301 bladder cancer cells (2 × 105 cells/200 μl DMEM) were plated in the upper chamber in the presence of 500 nM siRNA for c-Met, Axl and PDGFR-α, respectively, and analyzed for cell migration at 36 h. The filter was stained with 2% crystal violet, and the cells migrated to the opposite side of the filter were counted. (B) Quantification of (A). Data represents mean ± SD of three experiments. * *: p < 0.01, * * *: p < 0.001, by Student's t test. (C) The inhibitory effect of siRNA on c-Met, Axl and PDGFR-α expression was analyzed by Western blot analysis. Expression of c-Met, Axl and PDGFR-α was suppressed only in those cells transfected with c-Met, Axl or PDGFR-α siRNA/shRNA. There was no reduction of c-Met, Axl or PDGFR-α proteins in cells transfected with scrambled siRNA/shRNA. Ctrl: control treatment; S: scramble siRNA.

Upon HGF stimulation, c-Met induces several biological responses that collectively give rise to a program known as "invasive growth". To clarify the biological relevance of cross-talk among c-Met, Axl and PDGFR-α, cell migration assay was conducted. The transwell experiment showed that migration of TSGH8301 bladder cancer cells was considerably suppressed by c-Met siRNA knock-down (p < 0.001). In addition, apparent inhibition was also demonstrated when shRNA for Axl or PDGFR-α alone was treated (p < 0.01) (Figure 5A and 5B). Figure 5C shows that the siRNA for c-Met and shRNAs for Axl and PDGFR-α,TSGH8301, indeed suppressed their target gene expression in TSGH8301 cells. This result is consistent with the reports on Axl in the breast [37] and liver cancers [38], and on the PDGFR-α in liver cancer [39], respectively. Our result suggests that c-Met, Axl and PDGFR-α may induce comparable biological functions, possibly through the same signaling pathway or inter-connecting signal network.

### Clinical implication of c-Met, Axl, and PDGFR-α co-expression patterns in human bladder cancer patients

**Figure 6:**
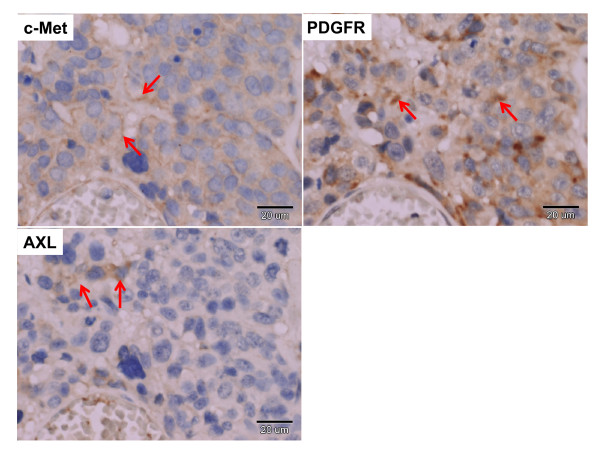
**Co-expression of c-Met, Axl and PDGFR-α in a human bladder cancer specimen**. Representative results of co-expression of c-Met, Axl and PDGFR-α in three serial sections of a human bladder cancer specimen. The three sections were incubated with anti-c-Met, anti-Axl or anti-PDGFR-α antibody, respectively (200X). The arrow indicates the specific expression of receptor protein around the membrane of the cancer cells.

**Figure 7:**
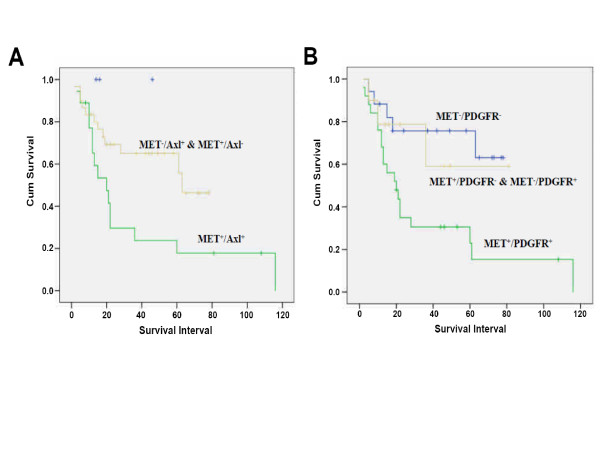
**Prognostic significance of co-expression of c-Met and Axl or PDGFR-α in human bladder cancer patients**. Kaplan-Meier survival analysis revealed that co-expression of c-Met/Axl (A) or c-Met/PDGFR-α (B) is significantly associated with poor survival in bladder cancer patients than those with single-receptor-positive or no receptor expression (*p *= 0.021 & 0.049, respectively).

To clarify the clinical implication of the above-mentioned findings *in vitro*, expression levels of c-Met, Axl and PDGFR-α were examined by immunohistochemistry in a total of 65 cases of locally advanced and metastatic bladder tumors. Co-expression of c-Met/Axl/PDGFR-α in a case of a bladder cancer tissue was demonstrated in Figure 6. Collectively, overexpression of c-Met, Axl, and PDGFR-α was found in 30 (46.2%), 52 (80%), and 40 (61.5%) cases, respectively. Co-expression of two receptors was revealed in 22 (33.8%, c-Met/Axl), 27 (41.5%, c-Met/PDGFR-α), and 17 (26.2%, Axl/PDGFR-α) cases. Fourteen cases (21.5%) showed co-expression of three receptors (c-Met/Axl/PDGFR-α). In these human bladder tumors, over-expression of PDGFR-α was correlated with nodal metastasis and overexpression of c-Met or c-Met/Axl/PDGFR-α showed the most significant correlation with poor patient survival (*p *< 0.01) followed by c-Met/PDGFR-α, PDGFR-α, c-Met/Axl, and Axl/PDGFR-α (*p *< 0.05) (table 2). Kaplan Meier survival analysis showed that cumulative survival of patients with high expression of c-Met/Axl and c-Met/PDGFR-α was significantly lower than those with lower expression (Log rank test, *p *< 0.05) (Figure 7A and 7B). After adjusting for nodal status, multivariate analysis using log rank test revealed that indicators associated with poor long term survival were over-expression of c-Met and co-expression of c-Met/Axl/PDGFR-α (*p *= 0.015) (data not shown). We next used a Cox proportional hazards models to determine the relative risk (RR) of overall survival with 95% confidence interval (CI). The RR of poor long term survival was 3.340 for over-expression of c-Met, and 3.860 for co-expression of c-Met/Axl/PDGFR-α. Taken together, our results indicate that, in addition to c-Met, both Axl and PDGFR-α play a positive role in the progression of human bladder cancer.

**Table 2:** Correlation of c-Met, Axl, and PDGFR-α protein expression with clinicopathologic parameters of patients with locally advanced and metastatic bladder cancers

Expression pattern	Grade	T status*	Multiple	Node (+)	Survival
c-Met	0.561	0.904	0.727	0.321	0.009^†^
Axl	0.409	0.105	0.795	0.300	0.789
PDGFR-α	0.344	0.470	0.718	0.049^†^	0.027^†^
c-Met & Axl	0.140	0.070	0.277	0.061	0.031^†^
c-Met & PDGFR-α	0.184	0.686	0.957	0.802	0.011^†^
Axl & PDGFR-α	0.439	0.585	0.762	0.369	0.049^†^
c-Met & Axl & PDGFR-α	0.595	0.377	0.346	0.281	0.008^†^

*Statistical analysis was performed between stage pT2 (21), pT3 (27) and stage pT4 (17), according to TNM (2002) classification criteria.

^† ^*p *value less than 0.05.

## Discussion and conclusions

In this study, we showed that both Axl and PDGFR-α have a functional interaction with c-Met *in vitro *and *in vivo*. This is the first report showing their potential clinical importance in human bladder cancer. The results concur with co-expression of c-Met/PDGFR-α in all of 9 human bladder cancer cell lines reported by Black and his colleagues [40]. The interaction between c-Met and Axl or PDGFR-α was further corroborated by HGF stimulation and siRNA silencing experiments *in vitro*. The interaction among these three RTKs may be initiated by protein-protein interaction or signaling transduction. The former possibility was excluded by co-immunoprecipitation assay (data not shown). In terms of signal regulation, the successful inhibition of c-Met activation by PD98059, but not by FTI-277 (ras inhibitor) or PP2 (Src inhibitor), suggests a *ras*- and Src-independent MEK/ERK 1/2 signaling in the transactivation of Axl and PDGFR-α. Our results seem to imply the existence of a novel mechanism by which c-Met transactivates the expression of Axl and PDGFR-α. Additional experiments are required to clarify whether protein kinase C is involved in this cross-talk *in vivo*.

Further support for our hypothesis of regulation at transcriptional level comes from several prior reports. The Sp1/Sp3 cis-acting elements were demonstrated to activate the promoter of Axl in various cancer cell lines [41]. Moreover, Sp1 response elements are detected in PDGFR-α promoter region [42,43]. Given that c-Met induces the phosphorylation of Sp1 and enhances downstream gene expression through MEK/ERK signaling pathway [44,45], c-Met might up-regulate the expression of Axl and PDGFR-α through Sp1. The dose-dependent suppression of Sp1, Axl and PDGFR-α by c-Met siRNA supports our speculation (Additional file 2).

It has been reported that HGF is expressed in fibroblast-like cells, smooth muscle cells, and endothelial cells of the bladder [46]. Expression of c-Met on the cancer cell surface thus may enable the paracrine activation *in vivo*, irrespective of their capability to synthesize HGF. The correlation of co-expression of two or three of the RTKs with patient survival supports the "invasive growth" program in carcinomas with multiple RTK over-expression [13,15,47]. The prognostic significance of c-Met, whether alone or co-expressed with Axl/PDGFR-α, supports the clinical relevance of c-Met-directed therapy (e.g. PHA665752) for human bladder cancer. Since the importance of co-targeting therapy for human bladder cancer having co-expressed RTKs has been demonstrated [15,47], a prospective study is imperative to clarify the significance of Axl and/or PDGFR-α as an additional biomarker or implementation of MEK1/2 inhibitor in the design of c-Met-targeting therapy for human bladder cancer patients.

It is interesting to note that induction of Axl via ''kinase switching'' confers the Gleevec resistance in relapsed patients with c-Kit- or PDGFR-α-driven tumors of the gastrointestinal tract [48]. Therefore, evaluation of RTK expression profile in human cancer may provide signaling network information and help in prediction of potential drug resistance [49]. Olaussen *et al. *showed that combinations of tyrosine kinase inhibitors could induce a synergistic antitumor effect and thus improve the therapeutic efficacy [50]. When more highly selective or multi-target tyrosin kinase inhibitors become available, the discovery of co-expression of RTKs in cancer cells highlights the necessity for individualized therapies in the future.

## Competing interests

The authors declare that they have no competing interests.

## Authors' contributions

CYT, SMS, and HHY participated in conceptualization, carried out this study, and drafted the manuscript; JWS, YCL, JHC, SMT, GR and TYC participated in the microarray data collection and analysis; CHS and NHC provided the clinical specimens; NHC and HSL conceived of the study, and participated in its design and coordination. All authors read and approved the final manuscript

## Pre-publication history

The pre-publication history for this paper can be accessed here:

http://www.biomedcentral.com/1471-2407/11/139/prepub

## Supplementary Material

Additional file 1 **c-Met expression in NIH/3T3 and NIH-Met5 cell lines in the presence or absence of Tet and/or HGF**. The cells and the treatment is the same as Figure 1A, except the expression of IgG was shown as the loading control. M: protein marker. Click here for file

Additional file 2 **The relationship among c-Met, Sp1, Axl and PDGFR-α demonstrated by c-Met siRNA**. NIH-Met5 cells (1 × 10^6^/plate) were transfected with c-Met siRNA (250 nM and 500 nM) for 24 h. Then, cells were harvested and total protein was extracted and analyzed for c-Met, Sp1, Axl and PDGFR-α expression by Western blotting. β-actin was used as the internal control. The numbers under each band represent the relative intensity. Click here for file
