# Supplementary figures and images for: Transcriptional activation of the Axl and PDGFR-α by c-Met through a ras- and Src-independent mechanism in human bladder cancer
Source: BMC Cancer. 2011 Apr 16;11:139. doi: 10.1186/1471-2407-11-139 (PMC3101176; doi:10.1186/1471-2407-11-139)

## NIH-Met5

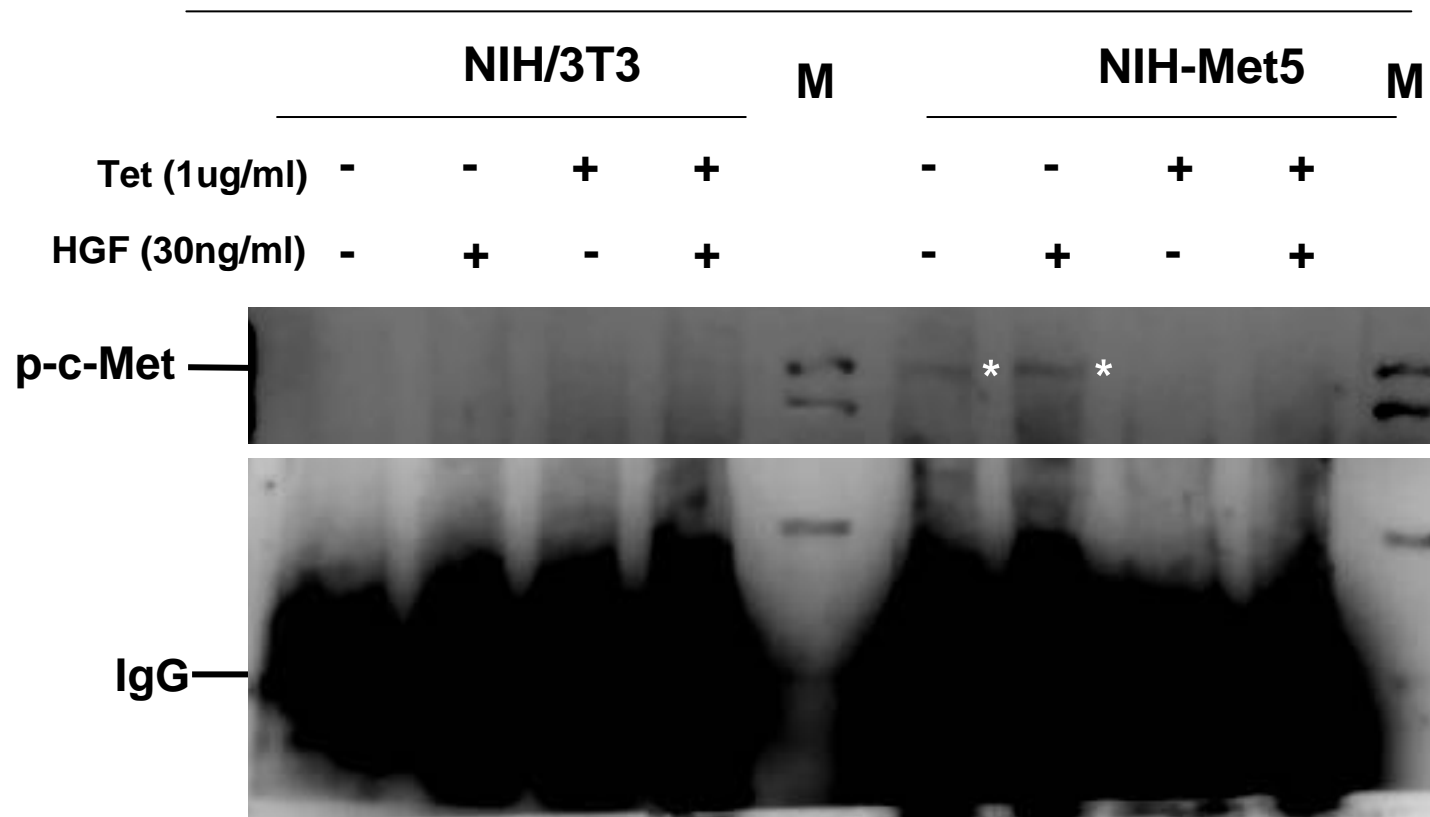

IP: phosphotyrosine

IB: c-Met

Supplement: Additional file 1 — c-Met expression in NIH/3T3 and NIH-Met5 cell lines in the presence or absence of Tet and/or HGF. The cells and the treatment is the same as Figure 1A, except the expression of IgG was shown as the loading control. M: protein marker. [file 1471-2407-11-139-S1.PDF]

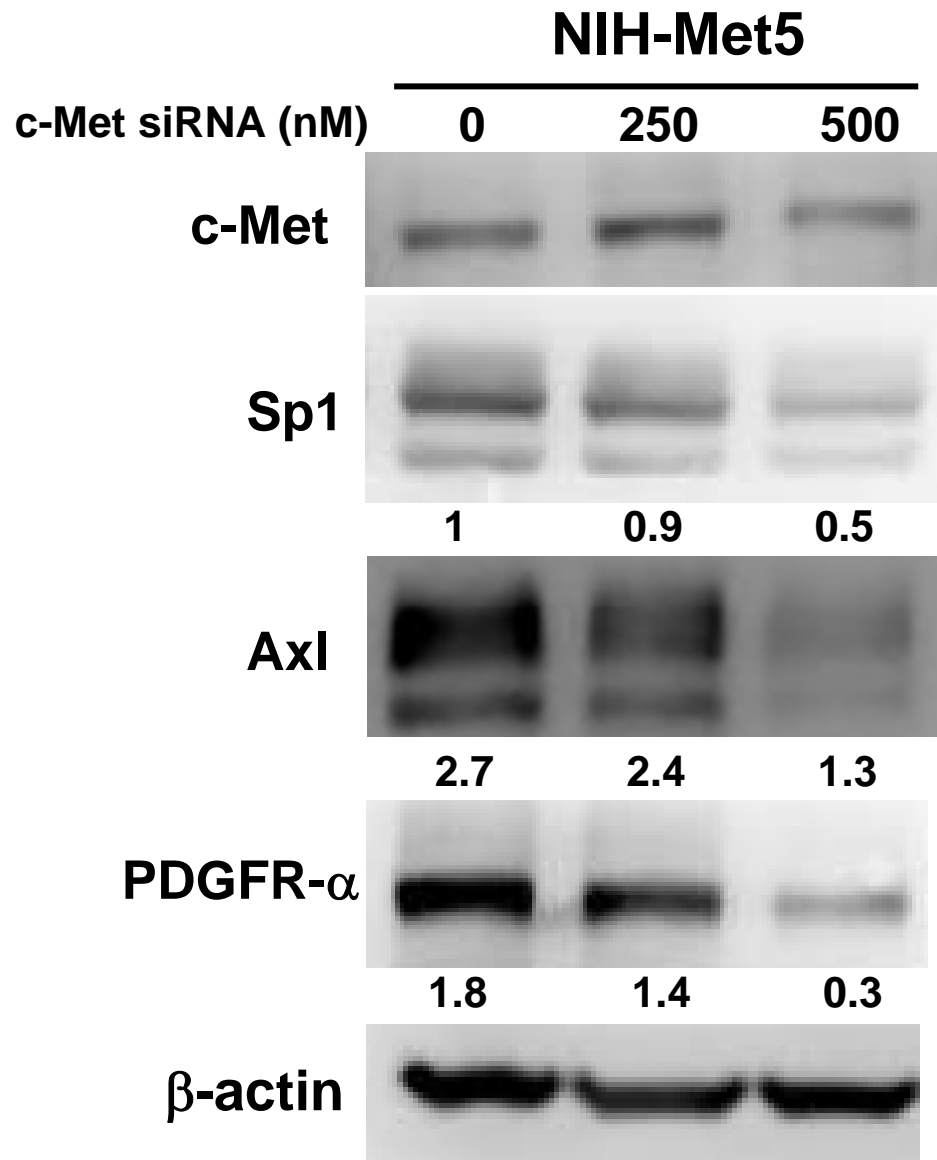

Supplement: Additional file 2 — The relationship among c-Met, Sp1, Axl and PDGFR-α demonstrated by c-Met siRNA. NIH-Met5 cells (1 × 10^6^/plate) were transfected with c-Met siRNA (250 nM and 500 nM) for 24 h. Then, cells were harvested and total protein was extracted and analyzed for c-Met, Sp1, Axl and PDGFR-α expression by Western blotting. β-actin was used as the internal control. The numbers under each band represent the relative intensity. [file 1471-2407-11-139-S2.PDF]
